# Optimal Surgery Type and Adjuvant Therapy for T1N0M0 Lung Large Cell Neuroendocrine Carcinoma

**DOI:** 10.3389/fonc.2021.591823

**Published:** 2021-03-24

**Authors:** Kunwei Peng, Huijiao Cao, Yafei You, Wenzhuo He, Chang Jiang, Lei Wang, Yanan Jin, Liangping Xia

**Affiliations:** VIP Region, Sun Yat-sen University Cancer Center, State Key Laboratory of Oncology in South China, Collaborative Innovation Center for Cancer Medicine, Guangzhou, China

**Keywords:** lobectomy, overall survival, lymph nodes dissection, adjuvant chemotherapy, large cell neuroendocrine carcinoma

## Abstract

**Background:**

The appropriate treatment strategy for T1N0M0 lung large cell neuroendocrine carcinoma (LCNEC) was not well illustrated. We evaluated the efficacy of different surgery types and adjuvant therapy on patients with T1N0M0 LCNEC.

**Methods:**

Patients diagnosed T1N0M0 LCNEC from 2004 to 2016 were identified in the surveillance, epidemiology, and end results (SEER) database. Clinical characteristics, treatment and survival data were collected. The efficacy of surgery type and adjuvant therapy stratified by tumor size was assessed. Overall survival(OS) was evaluated by the Kaplan-Meier method, and relevant survival variables were identified by the Cox proportional hazard model.

**Results:**

From 2004 to 2016, 425 patients were included in this study, 253 (59.5%) patients received lobectomy, and 236 (55.5%) patients had 4 or more lymph nodes removed. Patients received lobectomy had better survival than those received sublobar resection(*P*=0.000). No matter tumor size less than 2 cm or 2 to 3 cm, lobectomy was significantly prolonged survival. Compared with no lymph nodes removed, lymph nodes dissection was associated with more remarkable OS(*P*<0.000). 4 or more regional lymph nodes dissection predicted better OS compared with 1 to 3 regional lymph nodes dissection(*P*=0.014). After surgery, adjuvant chemotherapy did not contribute to extended survival in patients with tumor less than 2 cm(*P*=0.658), and possibly for tumor 2 to 3 cm(*P*=0.082). Multivariate analysis showed that age and lobectomy were independent prognostic factors(*P*=0.000).

**Conclusion:**

Our results suggest that lobectomy and lymph nodes dissection were associated with significantly better survival. Extensive regional lymph node dissection(4 or more) was more effective in prolonging survival than 1 to 3 lymph nodes dissection. Adjuvant chemotherapy was not associated with extended survival for tumor less than 2 cm, and possibly for tumor 2 to 3 cm.

## Introduction

Large cell neuroendocrine carcinoma (LCNEC) was traditionally classified as a subtype of non-small cell lung cancer (NSCLC), accounting for approximately 3% of all lung cancer (1, 2). However, it lacks specific histological characteristics of NSCLC, such as adenocarcinoma or squamous cell carcinoma, and presents neuroendocrine characteristics similar to small cell carcinoma (3). LCNEC usually expresses typical neuroendocrine tumor markers, such as chromogranin A, synapsin, CD56, neuron-specific enolase (NSE), these markers are of great significance in the diagnose of LCNEC (4).

LCNEC has a relatively high recurrence rate after surgery, the 5-year survival rate is only 40% even in early stage (5). Unfortunately, scarce prospective studies explored the optimal treatment on patients with LCNEC. The management of LCNEC was mainly referred to NSCLC and small cell lung cancer(SCLC). Like NSCLC, surgery was recommended for all patients with resectable disease (6). For stage I and stage II NSCLC, surgical resection was the primary treatment, with a 5-year survival rate of 60%-70%, 35%-40%, respectively (7, 8). The surgical methods include lobectomy, segmentectomy, and wedge resection. In addition, the thresholds of lymph nodes dissection have been proposed in NSCLC, range from 4 to 20 (9, 10). But, the indications of different surgery types for LCNEC have not been specifically illustrated. Adjuvant chemotherapy was widely accepted as a necessary treatment in lymph node positive NSCLC. The efficacy of adjuvant chemotherapy is unclear in lymph node negative tumors, except for some high-risk patients. Most studies suggest that postoperative adjuvant chemotherapy for stage I NSCLC patients could not improve the prognosis (11, 12), but in stage II NSCLC (13). Until now, the tumor size cutoffs for early stage LCNEC adjuvant chemotherapy and adjuvant radiotherapy have not been well studied.

LCNEC was considered as a specific solid tumor with poor prognosis. The appropriate choice of surgery type and adjuvant therapy was uncertain, particularly in T1N0M0 disease. Thus developing a consensus on optimal treatment of patients with T1N0M0 is necessary. In this study, we used data from the surveillance, epidemiology, and end results (SEER) database, to investigate the efficacy of different surgery type and adjuvant therapy on T1N0N0 LCNEC.

## Materials and Methods

### Patient Selection

The SEER database was supported by the National Cancer Institute (NCI), and providing information including cancer incidence, staging, and patient survival from 18 population-based cancer registries. Patients diagnosed with LCNEC according to the ICD-0-3/WHO 2008 from 2004 to 2016 were recruited. All patients were screened according to criteria as follows: 1) tumor located in “lung and bronchus”, pathological types was LCNEC; 2) tumor diagnosed from 2004 to 2016; 3) tumor diameter less than 3 cm, without lymph nodes and distant metastases; 4) patients who with the second primary tumor were excluded; and 5) patients without detailed information about surgery type were excluded. The primary endpoint of this study was OS. Demographic data included race, gender, age at diagnosis, and tumor differentiation.

### Statistical Analysis

Inter-relationships between variables were analyzed using the chi-square test or Fisher’s exact as appropriate. Cox proportional hazards regression was performed to assess risk factors. Variables with a *P* less than 0.10 on univariate regression analysis were subsequently examined in a multivariable model. Cumulative survival and differences were calculated using the Kaplan-Meier method and the log rank-test, respectively. *P* value <0.05 was considered statistically significant. Statistical analysis was performed using IBM SPSS (version 22.0).

## Results

### Patient Characteristics

A total of 4135 patients were diagnosed with LCNEC between 2004 and 2016, of which 425 patients were included in this study. The clinical characteristics of all patients were shown in **Table 1**. The proportion of women was 55.3%, and men were 44.7%. The majority of patients (85.9%) were white, with 10.8% were black, and 3.3% were other races. Lesions were mainly located in upper and lower lobe (64.0% and 28.5%, respectively), with a few in the middle lobe or other position (6.1% and 1.4%, respectively). 290 patients with pathological differentiation information, with 2 were well differentiated, 13 were moderately differentiated, 212 were poorly differentiated, and 64 were undifferentiated. Besides, patients with tumor size less than 2cm account for 59.5%, others were 2 to 3cm.

**Table 1:** Patient characteristics.

Characteristics	N=425 (%)
Age (years) <65 ≥65	168 (39.5) 257 (60.5)
Gender Male Female	190 (44.7) 235 (55.3)
Race White Black Other	365 (85.9) 46 (10.8) 14 (3.3)
Primary site Other Upper lobe Middle lobe Lower lobe	6 (1.4) 272 (64.0) 26 (6.1) 121 (28.5)
Pathological differentiation Well Moderate Poor Undifferentiated Unknown	2 (0.5) 13 (3.1) 212 (49.9) 64 (15.1) 134 (31.5)
Tumor size ≤2cm 2< ≤3cm	253 (59.5) 172 (40.5)
Surgery No surgery Lobectomy Sublobar resection Extended resection	44 (10.4) 253 (59.5) 110 (25.9) 18 (4.2)
Lymph node dissection None 1 to 3 regional lymph node removed 4 or more regional lymph node removed Others	90 (21.2) 73 (17.2) 236 (55.5) 26 (6.1)
Radiotherapy Yes No	22 (5.2) 403 (94.8)
Chemotherapy Yes No	74 (17.4) 351 (82.6)

### Patient Treatment

Among the 425 patients, most patients had surgery (lobectomy:59.5%, partial resection:25.9%, extended resection:4.2%), while 44 cases (10.4%) did not receive operation. 236(55.5%) patients performed regional lymph nodes dissection, with 4 or more lymph nodes removed. 73(17.2%) patients just removed 1 to 3 lymph nodes. 90(21.2%)patients had no lymph nodes removed. Chemotherapy was performed in 74 patients, and 22 patients had radiotherapy. Compared with chemotherapy group, patients treated with chemotherapy were younger (*P*=0.002, **Table 2**). Besides, no significant differences in gender, race, primary site, pathological differentiation, tumor size, surgery type, number of lymph node dissection were noted between groups with or without chemotherapy (**Table 2**).

**Table 2:** Patient characteristics by chemotherapy. N=425.

Characteristics	Chemotherapy(%)	Non-Chemotherapy(%)	*P*
Age(years) <65 ≥65	41 (24.4) 33 (12.8)	127 (75.6) 224 (87.2)	0.002
Gender Male Female	35 (18.4) 39 (16.6)	155 (81.6) 196 (83.4)	0.622
Race White Black Other	66 (18.1) 7 (15.2) 1 (7.1)	299 (81.9) 39 (84.8) 13 (92.9)	0.523
Primary site Other Upper lobe Middle lobe Lower lobe	1 (16.7) 41 (15.1) 6 (23.1) 26 (21.5)	5 (83.3) 231 (84.9) 20 (76.9) 95 (78.5)	0.389
Pathological differentiation Well Moderate Poor Undifferentiated Unknown	0 2 (15.4) 34 (16.0) 17 (26.6) 21 (15.7)	2 (100) 11 (86.4) 178 (84.0) 47 (73.4) 113 (84.3)	0.314
Tumor size ≤2cm 2< ≤3cm	37 (14.6) 37 (21.5)	216 (85.4) 135 (78.5)	0.066
Surgery No surgery Lobectomy Sublobar resection Extended resection	12 (27.3) 43 (17.0) 16 (14.5) 3 (16.7)	32 (72.7) 210 (83.0) 94 (85.5) 15 (83.3)	0.303
Lymph node dissection None 1 to 3 regional lymph node removed 4 or more regional lymph node removed Others	22 (24.4) 7 (9.6) 39 (16.5) 6 (23.1)	68 (75.6) 66 (90.4) 197 (83.5) 20 (76.9)	0.075
Radiotherapy Yes No	10 (45.5) 64 (15.9)	12 (54.5) 339 (84.1)	0.000

### Efficacy of Different Surgery Types on Survival

**Figure 1:**
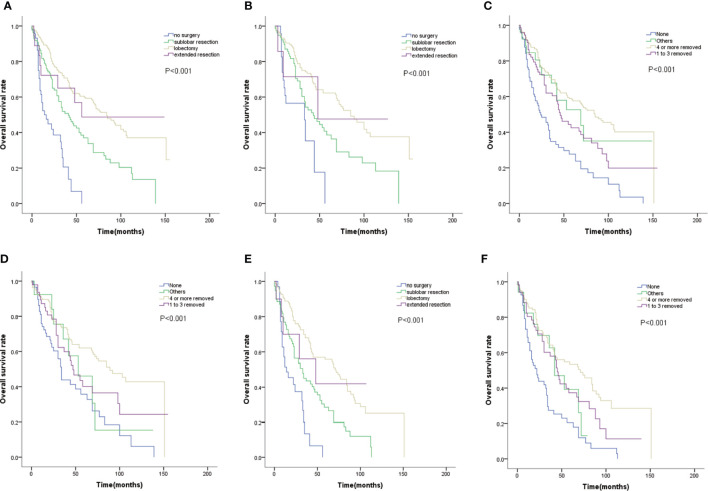
**(A)** OS for patients by surgery type. **(B)** OS for patients with tumor size smaller than 2cm by surgery type. **(C)** OS for patients by lymph nodes dissection type. **(D)** OS for patients with tumor size smaller than 2cm by lymph nodes dissection type. **(E)** OS for patients with age≥65 by surgery type. **(F)** OS for patients with age≥65 by lymph nodes dissection type.

We examined the efficacy of different surgery types on OS. Comparing with non-surgery group, patients with lobectomy, sublobar resection, or extended resection had better OS(*P*=0.000, **Figure 1A**). Moreover, patients treated with lobectomy had better outcomes than those treated with sublobar resection, with higher 3-year and 5-year survival rate(70.7% and 59.3% VS 51.9% and 37%; **Figure 1A**). Lobectomy extended median OS with almost 43 months (84 months vs 41 months; **Figure 1A**). Even in patients with tumors smaller than 2cm, lobectomy still improved the survival(**Figure 1B**). Among the surgical method of no lymph nodes removed,1 to 3 lymph nodes removed, and 4 or more lymph nodes removed, the survival distributions showed that patients with 4 or more lymph nodes removed had better survival(*P*=0.000, **Figure 1C**). Patients with no lymph nodes removed had distinctly worse OS than those with just 1 to 3 lymph nodes removed(*P*=0.02, **Figure 1C**). As same as lobectomy, regional lymph nodes dissection also improved survival in patients with tumors smaller than 2cm (**Figure 1D**). Even though advanced age was a risk factor of surgical complications, older patients could still benefit from lobectomy and regional lymph nodes dissection(*P*=0.000, **Figures 1E, F**).

### Efficacy of Adjuvant Therapy on Survival

**Figure 2:**
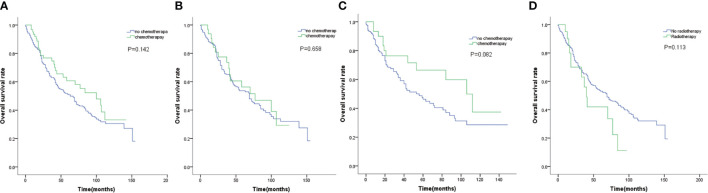
**(A)** OS for patients by chemotherapy. **(B)** OS for patients with tumor size smaller than 2cm by chemotherapy. **(C)** OS for patients with tumor size 2 to 3 cm by chemotherapy. **(D)** OS for patients by radiotherapy.

We further examined the efficacy of adjuvant chemotherapy and radiotherapy on survival. As shown in **Figure 2A**, adjuvant chemotherapy did not significantly improved survival(*P*=0.142). When stratified by tumor size, adjuvant chemotherapy did not contribute to prolonged survival for tumor size less than 2 cm(*P* =0.658, **Figure 2B**), and possibly for tumor size 2 to 3 cm(*P*=0.082, **Figure 2C**). Besides, patients who did not receive radiotherapy tended to have longer survival without statistical difference(*P*=0.113, **Figure 2D**).

### Multivariate Analysis of Survival

Univariate analysis showed that younger age, surgical resection and regional lymph nodes dissection were associated with favorable prognosis (both *P*<0.05, **Table 3**). Patients with lesions in the lung lobes had better outcomes than those with lesions in the main bronchus(both *P*<0.05, **Table 3**), but it was similar between the upper and lower lobes. Multivariate analysis showed that age was an independent prognostic factor on OS(HR=1.796, 95% CI 1.34–2.046, *P*=0.000, **Table 3**). Especially, lobectomy was an independent protective factor with decreased risk of death by 65.9% (HR =0.341, 95% CI 0.197–0.632, *P*=0.000, **Table 3**).

**Table 3:** Univariate and multivariate Cox regression analysis of prognostic factors influencing survival outcomes. N=381.

Features	UnivariateHR (95% CI)	*P*-value	MultivariateHR (95% CI)	*P* -value
Age(years) <65 ≥65	Reference 2.072 (1.559-2.752)	0.000	Reference 1.796 (1.34-2.046)	0.000
Gender Male Female	Reference 0.855 (0.661-1.106)	0.234		
Race White Black Other	Reference 0.913 (0.588-1.142) 0.879 (0.434-1.784)	0.687 0.722		
Primary site Others Upper lobe Middle lobe Lower lobe	Reference 0.275 (0.121-0.624) 0.390 (0.154-.978) 0.299 (0.129-0.-692)	0.002 0.047 0.005	Reference 0.409 (0.175-0.955) 0.627 (0.241-1.630) 0.399 (0.167-0.952)	0.039 0.338 0.038
Pathological differentiation Well Moderate Poor Undifferentiated Unknown	Reference 0.992 (0.122-8.094) 0.983 (0.137-7.043) 1.06 (0.145-7.758) 1.101 (0.153-7.929)	0.994 0.986 0.954 0.924		
Tumor size ≤2 2< ≤3	Reference 1.219 (0.939-1.582)	0.137		
Surgery No surgery Lobectomy Sublobar resection Extended resection	Reference 0.219 (0.146-0.329) 0.418 (0.274-0.638) 0.235 (0.107-0.514)	0.000 0.000 0.000	Reference 0.341 (0.187-0.632) 0.555 (0.335-0.918) 0.395 (0.161-0.971)	0.000 0.022 0.043
Lymph node dissection None 1 to 3 regional lymph node removed 4 or more regional lymph node removed Others	Reference 0.549 (0.386-0.799) 0.359 (0.263-0.489) 0.432 (0.238-0.783)	0.002 0.000 0.006	Reference 0.859 (0.539-1.369) 0.747 (0.464-1.203) 0.708 (0.638-1.360)	0.523 0.231 0.300
Radiotherapy Yes No	Reference 0.641 (0.379-1.084)	0.097	Reference 0.718 (0.419-1.231)	0.229
Chemotherapy Yes No	Reference 0.892 (0.632-1.29)	0.517		

HR, hazard ratio; CI, confidence interval.

## Discussion

Previously, only case report or small sample studies on LCNEC had been reported due to its scarcity (14). Therefore, the optimal treatment modality of early stage LCNEC has not been adequately established. In this work, we evaluated the role of different surgery types and adjuvant therapy on T1N0N0 LCNEC patients from the SEER database between 2004 and 2016. We found that lobectomy was strongly associated with better survival, even for patients with tumors smaller than 2 cm. Furthermore, patients who received 4 or more lymph nodes dissection had longer survival than those who did not receive lymph nodes dissection. This advantage can be found even in who had 1 to 3 lymph nodes removed. Lastly, adjuvant chemotherapy did not contribute to prolonged survival in patients with tumors smaller than 2 cm, and possible for patients with tumors 2 to 3 cm.

For early stage lung cancer, surgery such as lobectomy, segmentectomy, and wedge resection was recommended. In previous studies, patients undergoing sublobar resection tended to have increased local recurrence rate compare to lobectomy with no significant difference in OS (15–17). Thus, the choice of lobectomy or sublobar resection is controversial for stage I NSCLC. Study comparing different surgery types on limited stage SCLC indicate that, patients undergoing wedge resection experienced worse survival compared with those undergoing lobectomy; while similar results were observed between segmentectomy and lobectomy (18). In our study, lobectomy showed a greater advantage than sublobar resection on OS, suggesting that lobectomy was more appropriate for T1N0N0 patients regardless of tumor size. Some studies have shown that elderly patients have higher incidence of complications and mortality after lobectomy than young patients (19). In our study, patients selection bias may have been present, we found that lobectomy and regional lymph nodes dissection were feasible for older patients with survival significantly improved (P=0.000, **Figures 1E, F**).

On the premise of complete resection of tumor lesions, preserving the normal lung tissues of patients to the maximum extent and reducing surgical trauma has become the novel direction of surgery. Is lymph node dissection necessary for early stage patients? A large body of literatures supporting that lymph nodes dissection helps to prolong survival, even in early stage patients (20, 21). However, the optimal number of lymph nodes dissection remains unknown in T1N0M0 LCNEC. The threshold of lymph nodes dissection(4 or more) in our study consistent with other publications that focused only on stage I NSCLC (22, 23). Interestingly, removing 4 or more lymph nodes and removing 1 to 3 lymph nodes can both be used as prognostic indicators in the current study, which were our novel discovery compared to previous publication (24, 25). Current guidelines already recommend lobectomy and N1 and N2 resection for stage IA NSCLC, but the subjects were mainly NSCLC. Since LCNEC was a special type of NSCLC. This study focuses on the optimal surgery type for T1N0M0 LCNEC, and further confirmed that lobectomy and lymph node dissection were suitable for T1N0M0 LCNEC.

Adjuvant therapy for early stage LCNEC is debatable for years. The high risk of recurrence and the poor prognosis, drove efforts to identify patients who need postoperative adjuvant therapy. Some studies demonstrated positive results with respect to the benefit of adjuvant chemotherapy. A prospective study of stage I LCNEC suggested that adjuvant chemotherapy consisting of cisplatin and VP-16 for two cycles after surgical resection appeared promising improvement of prognosis (26). In another retrospective study, survival was prolonged in patients who received adjuvant chemotherapy in stage I LCNEC after surgery (27). Recent studies suggested that the role of adjuvant therapy for early-stage LCNEC remains to be determined, especially in tumor smaller than 3 cm (28). Even though in randomized prospective trials, the benefit of adjuvant chemotherapy was controversial for stage I NSCLC (12, 29). However, these studies were majorly concentrated on stage T2N0M0. In this study, adjuvant chemotherapy may not be necessary. The survival analysis failed to identify the benefit of adjuvant chemotherapy. On details, adjuvant chemotherapy was not associated with extended survival for tumor less than 2 cm, and possibly for tumor 2 to 3 cm. Adjuvant chemotherapy was recommended to all resected SCLC patients regardless of tumor size (30), as is NSCLC patients with high risk or tumor bigger than 4 cm (7, 8). Therefore, researches are needed to design stratifying by tumor size, which may be more applicable to LCNEC clinical practice. In the whole cohort, adjuvant radiotherapy was not associated with prolonged survival, and may be detrimental in terms of OS actually. The role of adjuvant radiotherapy was extensively investigated in lymph nodes positive patients. Assessment of National Cancer Database showed that, the use of adjuvant radiotherapy was associated with significantly improved OS in SCLC patients with pN2 disease, but a deleterious effect with pN0 disease (31). Due to the small number of patients receiving adjuvant radiotherapy, it is hard to identify the efficacy of adjuvant radiotherapy on tumor of different size. Whether adjuvant radiotherapy is necessary for postoperative patients needs to be investigated.

Similar to other researches using SEER database(PMID: 32500023, PMID: 31302755), this study has some limitations. Firstly, this is a retrospective study with a relatively large sample of T1N0M0 patients, some clinical features were not detailed enough, so the conclusions may have a bias. For example, the absence of pathological differentiation data in 120 patients limited our ability to analysis its predictive efficacy. Unfortunately, this is a database-based analysis, and we can’t get the tumor tissue, a pathological review is difficult. According to the traditional understanding, T1N0M0 patients are treated with surgery first and do not need adjuvant therapy, so the subgroups submitted to adjuvant and non-surgical treatment is relatively low. Secondly, we also lack information about chemotherapy regimens, making it difficult to evaluate the potential advances of postoperative adjuvant chemotherapy. Finally, the disease-free survival time is an important criterion for evaluating surgical methods and was not available from SEER database.

Since LCNEC was rare, and the treatment of LCNEC was simulated as the regimen of NSCLC and SCLC. We performed a population-based analysis, and the results showed that lobectomy was an independent protective factor. Besides, we observed that the number of regional lymph nodes removed significantly affected survival. Postoperative adjuvant therapy was not able to prolong survival. This is the largest study discussing treatment and outcome of T1N0M0 LCNEC, providing clues to understand the treatment and conduct prospective studies of T1N0N0 LCNEC.

## Data Availability Statement

Publicly available datasets were analyzed in this study. This data can be found here: Surveillance, Epidemiology, and End Results (SEER) database (https://seer.cancer.gov/).

## Ethics Statement

This study was approved by the institutional review board and ethics committee of Sun Yat-sen University Cancer Center. It was determined to be a retrospective analysis of publicly available data, and written informed consent was waived.

## Author Contributions

KP: original idea, data acquisition and analysis, and manuscript writing. HC: data acquisition and analysis, and prepared the figures and tables. YY: data analysis and manuscript editing. WH, JC, LW, and YJ made comments and manuscript editing. LX: project development and manuscript editing. All authors contributed to the article and approved the submitted version.

## Funding

This work was supported by grants from the Science and Technology project of Guangdong Province (2017A020215031) and Guangdong Medical Science and Technology Research Fund (C2018063).

## Conflict of Interest

The authors declare that the research was conducted in the absence of any commercial or financial relationships that could be construed as a potential conflict of interest.
